# Instagram Likes for Architectural Photos Can Be Predicted by Quantitative Balance Measures and Curvature

**DOI:** 10.3389/fpsyg.2018.01050

**Published:** 2018-06-22

**Authors:** Katja Thömmes, Ronald Hübner

**Affiliations:** Cognitive Psychology, Department of Psychology, Universität Konstanz, Konstanz, Germany

**Keywords:** visual aesthetics, Instagram Likes, aesthetic appeal, computational aesthetics, architectural photography, visual balance, image composition, curvature

## Abstract

“3,058 people like this.” In the digital age, people very commonly indicate their preferences by clicking a Like button. The data generated on the photo-sharing platform Instagram potentially represents a vast, freely accessible resource for research in the field of visual experimental aesthetics. Therefore, we compiled a photo database using images of five different Instagram accounts that fullfil several criteria (e.g., large followership, consistent content). The final database consists of about 700 architectural photographs with the corresponding liking data generated by the Instagram community. First, we aimed at validating Instagram Likes as a potential measure of aesthetic appeal. Second, we checked whether previously studied low-level features of “good” image composition also account for the number of Instagram Likes that architectural photographs received. We considered two measures of visual balance and the preference for curvature over angularity. In addition, differences between images with “2D” vs. “3D” appearance became obvious. Our findings show that visual balance predicts Instagram Likes in more complex “3D” photographs, with more balance meaning more Likes. In the less complex “2D” photographs the relation is reversed, more balance led to fewer Likes. Moreover, there was a general preference for curvature in the Instagram database. Together, our study illustrates the potential of using Instagram Likes as a measure of aesthetic appeal and provides a fruitful methodological basis for future research.

## Introduction

One aim of researchers in the field of experimental aesthetics is to investigate how our aesthetic preferences influence our daily decisions. There is no doubt that visual aesthetic properties exert considerable influence on our actions. This holds for both consumers—when they buy artfully designed products, enjoy visits to museums, galleries and exhibitions, or even search for an attractive partner—and for producers—when artists create artworks, advertisers design campaigns, researchers visualize data, or ordinary people arrange their flats, take photos, or do handicrafts. We all strive to put things together in a visually pleasing way, because what is beautiful is usually considered as good. Extensive research on the “beautiful is good” phenomenon has shown that it applies to persons—“who is beautiful has more socially desirable personality traits and leads a better life” (e.g., Dion et al., 1972; Eagly et al., 1991)—to product design and user interfaces—“what is beautiful is usable and works better” (e.g., Tractinsky et al., 2000; Norman, 2002)—and even to the perceived truth of scientific findings and theories—“what is beautiful is true” (e.g., Fischer in Krohn, 2006). This subjectively added value through beauty or aesthetic appeal is what motivates researchers to investigate fundamental aesthetic principles. A major challenge in this respect is to validly assess beauty and to explain its fundamentals.

In his seminal work *Aesthetics and Psychobiology* Berlyne states that “as far as aesthetics is concerned, the experimental psychologist or psychobiologist must concentrate on the scientific study of *aesthetic behavior*” (Berlyne, 1971, p. 7). Aesthetic behavior is observable in both creatives and performance artists, and in the “appreciator,” a term that Berlyne uses for a person who is exposed to a work of art (Berlyne, 1971, p. 7). Thus, if we want to learn something about the aesthetic appeal of photographs and its determinants, one way to do so is to observe the appreciators' aesthetic behavior. The aim of the present paper is to test the hypothesis that Instagram Likes reflect aesthetic behavior of thousands of online users—and that the total amount of Likes is determined, at least to some degree, by objective features of the uploaded images.

Specifically, we investigated whether the number of Likes on Instagram can be used as proxy for explicitly measured aesthetic preferences. Furthermore, we tried to uncover links between Instagram Likes and measures of low-level image properties. An important and basic property in this respect is visual balance, i.e., how well an image is composed (Ross, 1907; Arnheim, 1971, 1982). There are two prominent candidates related to this concept: the spatial distribution of perceptual “weight” or “mass” (cf. APB measure by Wilson and Chatterjee, 2005) and the location of the center of “mass” (cf. CoM by Bauerly and Liu, 2006; McManus et al., 2011). Both measures assume that the distribution of “mass” in a picture is determined by the distribution of luminance. Over the last years, several formal measures for these concepts have been developed (Bauerly and Liu, 2006; Hübner and Fillinger, 2016) and applied to simple geometric patterns (Wilson and Chatterjee, 2005; Hübner and Fillinger, 2016), portraits (Aleem et al., 2017), art and random photographs (McManus et al., 2011), and user interfaces (Ngo et al., 2002). In our study, we wanted to generalize the validity of these measures to architectural photographs. In addition, we examined whether the proposed aesthetic preference for curvature over angularity (e.g., Silvia and Barona, 2009) also holds for our selection of architectural images.

In the following, we first describe general insights into the aesthetic appeal of photographs and set out why Instagram might be used as a source of data in the field of empirical aesthetics. We then briefly introduce the considered image properties and their connections to aesthetic preferences.

### The aesthetic appeal of photographs and Instagram likes

In 1841, William Henry Fox Talbot, one of the “fathers” of photography, patented his technology of banning images on film. He named it Calotype, from the Greek word *kalos*, which means beautiful (Sontag, 1977, p. 84). It thus appears that from the very beginning, one primary objective of photographers was to create something beautiful. This makes photographs a promising object of investigation in the field of empirical aesthetics. This especially holds since large databases have become available on photo-sharing platforms such as Instagram. In September 2017, Instagram reached 800 million monthly active users, with 500 million users using the platform every day (Systrom, 2017). One key element of online photo-sharing platforms is the possibility for every user to express their preference for each image by clicking the “Like” button. For people who upload photographic content on their Instagram account, this is a great opportunity to get fast and direct feedback from the community. Although it can be assumed that anybody who shares his or her work on Instagram wants to receive as many Likes as possible, there is a marked difference between private persons and professional photographers. Whereas the former typically share selfies and snapshots from their daily life to communicate with friends, the latter use the platform to promote their professional work.

For the purposes of this study, we focus on professional photographers to minimize potential interference from social effects such as personal sympathy between friends. Of course, this does not eliminate potential effects of marketing efforts of the professionals. Success on Instagram depends on good knowledge of your target audience, a consistent theme and extensive content uploads (Carroll, 2017). However, marketing efforts should only affect the total number of Likes generated by a photographer, but not the variance between photos by the same photographer. Instagram users scrolling through their feed, press the “Like” button to appreciate the uploaded pictures. In most cases, clicking the Like button presumably happens rather intuitively without much thought, while scrolling through the Instagram feed, where users see all new uploads of people they follow. If it can be shown that the number of Likes corresponds to the aesthetic appeal of photographs, this would offer novel approaches for studying principles of aesthetic preference.

**Figure 1:**
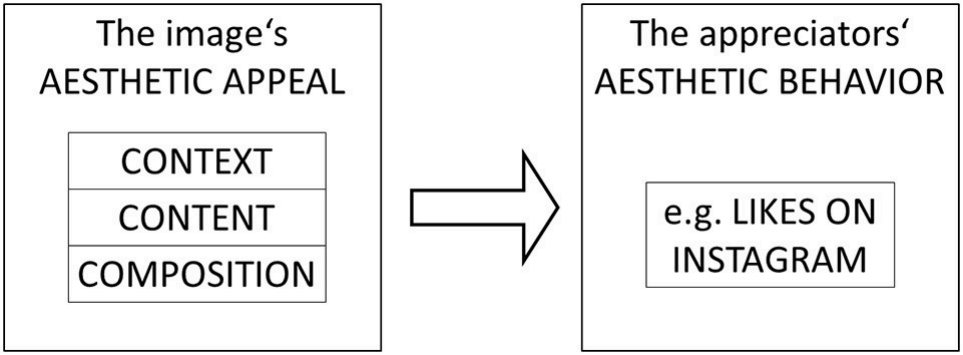
The aesthetic appeal of an image is determined by its content, its composition, and the context. Instagram Likes are interpreted as aesthetic behavior in the sense of Berlyne (1971) that are tied to the aesthetic appeal of an image.

So, what determines the aesthetic appeal of an image? In the case of photographs as objects of aesthetic interest, aspects that determine their aesthetic appeal fall into one of three categories: content (What is depicted?), context (e.g., What information, such as text or titles, accompany the image?), and composition (How is the photograph composed?). For a successful investigation of the extent to which formal features of the composition determine aesthetic responses, it is important to be aware of confounding effects of content and context and try to control for them. Figure 1 illustrates the scheme.

With respect to content, anecdotal evidence suggests that uploading photos of babies or kittens increases the number of Likes from the community. Bakhshi et al. (2014) analyzed a database of one million Instagram posts, and found that photos containing human faces were 38% more likely to receive Likes (Bakhshi et al., 2014). Thus, to control for large effects of content, it is important to compare photographs with similar content. In the present study, we restricted our analyses to architectural photographs posted by five different photographers.

With respect to context, there is evidence that titles and additional information texts influence the aesthetic appeal of an image (Leder et al., 2006; Thömmes and Hübner, 2014). Thus, when it comes to online photo sharing platforms it is important to figure out which contextual factors affect people's aesthetic experience when looking at photos on digital screens. On Instagram, the use of hashtags is a crucial point, as those tags contextualize the photographs and at the same time provide the opportunity to find other images with the same hashtag. Also, it is likely that the timing of the post (time of the day, week vs. weekend), the country or city where the photo was taken, and current trends influence the number of Likes. On our search for architectural photographs for the Instagram database we used the hashtags *#architecture* and *#minimalarchitecture*.

This paper aims at uncovering the significance of certain aspects of composition. There are different compositional features that are likely to affect the aesthetic appeal of photographs, such as symmetry, balance, lines and forms, contrasts, colors, golden ratios, etc. Our goal is to examine the relationship between several quantitative and qualitative measures of image features related to composition and the number of Likes. Importantly, for photographs composition is also related to image format. There are various possibilities of framing and cropping compositions, including wide panoramic images, standard rectangular formats in portrait or landscape orientation, and square format. In the present study, though, we control for format by using only quadratic images (the prototypical Instagram format) and investigate effects of compositional features regarding balance and curvature.

### Formal low-level features and their aesthetic appeal

What makes good composition has long been an object of study in different fields of visual arts, such as graphic design, painting, and photography. Several approaches exist to get to the basics accounting for good composition from a computational point of view. A general method for optimizing photo composition targets the rule of thirds, diagonal dominance, and visual balance as basic aesthetic guidelines (Liu et al., 2010). There is evidence that such compositional features are connected to the aesthetic appeal of images, as we will describe below. Of the many formal properties, visual balance has received much attention (Wilson and Chatterjee, 2005; McManus et al., 2011; Hübner and Fillinger, 2016). We will describe current findings on aesthetics and visual balance, divided into studies on symmetry and balance, as well as the preference for curvature.

#### Preference for symmetry

Symmetry is the simplest form of balance (Ross, 1907; Wilson and Chatterjee, 2005). It is well known that symmetric objects are preferred (e.g., Weyl, 1980; Palmer, 1991; Ramachandran and Hirstein, 1999) and that this preference already exists in infants (Humphrey and Humphrey, 1989). Studies have confirmed the preference for symmetry with respect to geometric forms and polygons (Jacobsen and Höfel, 2002), as well as facial painting and abstract design (Cárdenas and Harris, 2006). Symmetry is also associated with facial attractiveness (Rhodes et al., 1998), which might be due to symmetry's association with biological fitness and health (Thornhill and Gangestad, 1999; Rhodes, 2006). Symmetry accounts for faster processing and better memorability (Garner and Clement, 1963) and is even implicitly connected to positive attributes (Makin et al., 2012). A prominent account of symmetry preference is perceptual fluency (Winkielman et al., 2003; Reber et al., 2004). It is assumed that fluent sensory processing elicits positive feelings, which in turn enhance aesthetic pleasure. Indeed, there is evidence that symmetrical patterns are processed faster than non-symmetrical ones (Makin et al., 2012). Locher and Nodine (1989) claim that the rapid detection of symmetry by the perceptual system reflects a fundamental unlearned process.

#### Preference for balance

In a conceptual sense, perfect balance is a state, where all opposing forces equilibrate in a point of perfect stillness as compared to the perception of movement, once balance is lost (Ross, 1907, pp. 1–2). Highly balanced compositions do not necessarily have to be symmetric. The conceptual vocabulary in the relevant literature includes the terms complex symmetry, balance, or even harmony (Puffer, 1903; Ross, 1907; Arnheim, 1982). Photographs with clearly asymmetric compositions can still be perfectly equilibrated and therefore harmonious, for instance when smaller dark areas are moved farther from the center than larger dark areas on the opposite side (Samuel and Kerzel, 2013). The brain seems to have special mechanisms for processing balance, as suggested by the finding that absence of balance leads to the need of attention (Itti et al., 1998). Despite the long debate, quantifying balance and finding links between aesthetic appeal and compositional balance is an ongoing challenge. Results from early studies in which subjects judged balance by placing a horizontally adjustable fulcrum beneath an image (Monroe, 1925; McManus et al., 1985) suggest that the distribution of perceptual “weight” based on luminance is a crucial factor for perceived balance. Dark areas in a composition are perceived as heavier than bright areas. Accordingly, most current measures are based on this analogy to physical balance (e.g., Wilson and Chatterjee, 2005; Bauerly and Liu, 2006). It should also be mentioned that there is research focusing on visual saliency or attention as the issue for perceived balance (e.g Abeln et al., 2015; Jahanian et al., 2015) which, however, will not be dealt with in detail here. Luminance-based measures of pictorial balance have been applied to different visual stimuli, such as compositions of black geometrical shapes on a white background (Wilson and Chatterjee, 2005; Hübner and Fillinger, 2016), Japanese calligraphy (Gershoni and Hochstein, 2011), screen layouts in web design (Ngo et al., 2002), Renaissance paintings (Aleem et al., 2017), art photographs (McManus et al., 2011), and Flickr photographs (Schifanella et al., 2015). Most of the corresponding studies show that the objective measures correlate with subjective ratings of balance. To the best of our knowledge, there have been only few studies investigating whether these measures also predict aesthetic preference. Of these, some find empirical support for this hypothesis (Wilson and Chatterjee, 2005; Hübner and Fillinger, 2016), whereas others yield inconsistent results (McManus et al., 2011).

#### Preference for curvature over angularity

Curvature as a physical property of visual objects has been investigated for pictures of real objects such as sofas and watches, as well as for meaningless patterns. For all stimuli the preference for curved over angular objects is present (Bar and Neta, 2006). Even when controlling for symmetry, balance, and typicality, meaningless patterns and polygons are preferred when they are curved rather than angular (Silvia and Barona, 2009). A positive effect of curvature is even found in the design of consumer products, where round contours increase the likelihood of purchase (Westerman et al., 2012). Visual preference for curvature is mostly explained by human evolution and is considered an aesthetic primitive. It is assumed that sharp contours convey some threat of harm leading to a negative bias (Bar and Neta, 2006). Support for the evolutionary hypothesis comes from the finding that humans share this preference with great apes, even though it is more evident in humans (Munar et al., 2015). There is also evidence for an association of curvature with positive attributes (Palumbo et al., 2015). Moreover, recent studies have shown that individual differences in expertise, personality, and cognitive style moderate the preference for curvature as opposed to angularity. Higher artistic expertise and general openness to experience predict greater preference for curvature (Cotter et al., 2017). Carbon (2010) found strong reliance of these curvature effects on Zeitgeist factors, as curvature and angularity in the design of cars change dynamically over time and so does appreciation.

## The Instagram database

For our Instagram database we compiled a corpus of around 700 architectural photographs in square format and their corresponding liking data. The photos have been published by five different Instagram accounts.

In architectural photography, the main challenge for the photographer arguably is good composition rather than conveying a certain situation, as for example in street photography. The photographs chosen for our database are deliberate compositional studies, which makes them especially suitable for examining the fundamentals of photographic composition. As mentioned above, it can be argued that the effects of low-level features will differ in different formats (Ross, 1907). Especially the importance of the center is greater in square frames than in rectangular ones, because the center of a square has the same relation to each of its four sides which is not true for any other rectangular format (Arnheim, 1982). Accordingly, many studies that have computed measures for low-level image features have used square format (e.g., Hübner and Fillinger, 2016). Therefore, we explicitly selected architectural photographers, who publish their work in square format on Instagram.

Because we wanted to analyze the number of Likes, we also had to take into consideration the context in which the Likes are generated. We established several criteria that photographers must fullfil to be included in our database. First, to ensure that a wide range of people see and rate the pictures by hitting the Like button (or not), we only chose Instagram accounts with a minimum of 10,000 followers. By selecting professional photographers with many followers, there should only be few socially motivated Likes (Jang et al., 2015). For professional photographers with a large followership we thus assume that most Likes are motivated by actual aesthetic liking. A legitimate objection is that people who follow professional architecture photographers are very likely to have specific preferences for this type of content. This limits the scope of our data, but we are convinced that the benefits of the enormous sample size due to large followerships outweigh the limitations of such “interested” samples.

**Figure 2:**
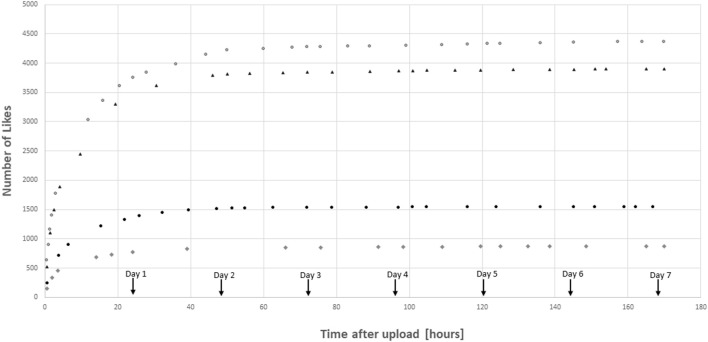
Examples from four posts on different Instagram accounts showing how the number of Likes typically emerges over time.

Another important issue is the influence of time passed after the upload of a photograph. Most Likes are given within the first couple of days or even hours after a photo is published. Figure 2 shows examples of the development of Likes for four different posts by different accounts. There is only little increase in the number of Likes for an individual photograph after it has been online for more than a week. The reason for this is most likely the enormous number of posts on Instagram every day. Users typically only see the most recently posted photographs unless they scroll down the time line of one particular Instagram account. The development of the number of followers over time is also an important factor. As every user starts out at zero followers, the first uploads of a photographer tend to have less Likes compared to later photographs. Therefore, it makes sense to look at the number of Likes for photographs published more than one week ago, while controlling for time effects due to a growing followership over time.

We collected the photographs using a software for fast and easy downloads from Instagram^1^ and gathered the corresponding number of Likes in late 2016. More details are found in the description of Table 1. The five photographers whose photographs are used in the Instagram database are also listed and described in Table 1. All photographers gave us permission to use their photographs for scientific purposes.

**Table 1:** This table describes the characteristics of the selected photographers (Instagram names in parentheses).

**Photographer**	**Content**	**Posting on Instagram since**	Number of Followers^*^	Number of published photos^*^	Number of selected photos^**^	Number of excluded photos^***^	**Photos in the database**	**Number of Instagram Likes**	
							***N***	**Mean**	***SD***
Alex Hamburg (fernsehturm_)	Close-ups, Surfaces	Nov 2016	16,000	980	245	1	244	1,108	336
Maik Lipp (usrdck)	Facades of buildings	Jan 2016	10,000	277	53	4	49	988	392
Matthieu Venot (matthieuvenot)	Facades of buildings	May 2013	84,000	495	61	–	61	1,073	210
Sebastian Weiss (le_blanc)	Facades of buildings	Dec 2010	180,000	705	278	10	268	3,094	672
Kai Ziehl (kaiziehl)	Cityscapes, silhouettes	Jul 2014	10,000	147	57	–	57	1,421	708
Total					694	15	679	

* *Data were collected in December 2016*.

** *We selected all posts that met the criteria within the following time frames: Alex Hamburg: Apr 16–Nov 16; Maik Lipp: May 16–Oct 16; Matthieu Venot: Oct 15–Jun 16; Sebastian Weiss: Feb 14–Nov 16; Kai Ziehl: Feb–Nov 16. In the case of Kai Ziehl, only photographs with one human silhouette were selected; photographs with e.g., couples holding hands, hugging each other, or other social situations were not selected*.

*** *15 images were excluded from analysis for one of the following reasons: no quadratic format, no architectural content, logos, or lettering on the buildings, extremely colorful compositions*.

**Figure 3:**
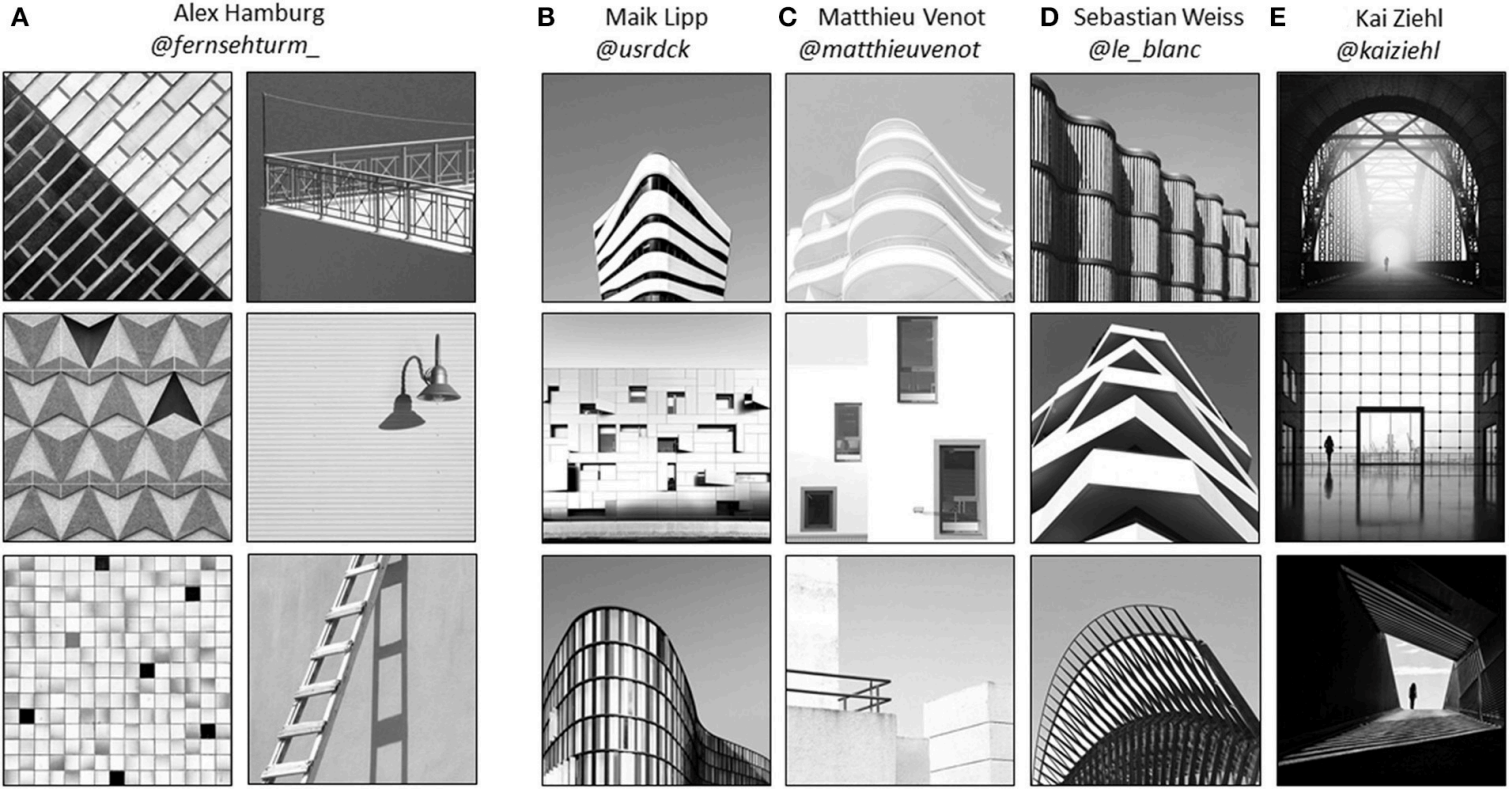
Example photographs included in the Instagram database arranged by “2D” vs. “3D” appearance. **(A)** Three examples of “2D” compositions: rotation-invariant close-ups. Three examples of Alex Hamburg's 3D compositions: not fullfilling the strict criterion of rotation-invariance. **(B–E)** Three examples of “3D” compositions for each photographer: facades of buildings and cityscapes. Note: Only Kai Ziehl originally posts his work in black-and-white, pictures by the other photographers were originally posted in color.

Notwithstanding the fact that we only use architectural photographs, it is useful to do a finer subdivision concerning the overall appearance of the images. As we wanted to use measures that were previously applied to very simple stimuli such as geometric forms and patterns, we tried to find very elementary photographic compositions as a starting point. The simplest compositions in the database are those by Alex Hamburg. He fills the frame solely with textures and patterns of different surfaces in the cityscape and therefore creates photographs that appear rather two-dimensional. For our purposes, we classified photographs as having a “2D” appearance based on rotation invariance, meaning that there is no recognizable top or bottom in the photograph. Only about half of Alex Hamburg's photographs (*n* = 132) withstand this strict criterion of being invariant to rotation, the other pictures are somewhat more complex with objects like stairs, street lamps or the sky in the composition and therefore classified as 3D. His photos were categorized as “2D” or “3D” by one of the authors (KT). The other photographers (Maik Lipp, Matthieu Venot, Sebastian Weiss, Kai Ziehl) produce more complex photographic compositions of the facades of buildings or cityscapes with clearly three-dimensional appearance. Their work can be seen as minimalistic compositional studies in architectural photography. Examples of photographs with “2D” vs. “3D” appearance are shown in Figure 3. It is important to note that the “2D/3D” classification is confounded with complexity (for a comprehensive overview of visual complexity cf. Gartus and Leder, 2017) and that some studies found an inverted U-shaped relation between preference and complexity (Berlyne, 1971; Imamoglu, 2000). People seem to prefer moderate levels of complexity compared to overwhelmingly high levels or underwhelmingly low levels. This preference might reflect the brain's search for a compromise between maximizing information intake and maintaining comprehensibility (cf. Aleem et al., 2017). However, there are also studies that have found evidence for rather linear relationships between complexity and aesthetic preference (Stamps, 2002; Nadal et al., 2010; Güçlütürk et al., 2016).

Our final database consists of 679 Instagram photographs with their corresponding numbers of Likes. You find descriptive statistics of the numbers of Likes per photographer in the right column in Table 1.

## Experiment 1: predicting two-alternative forced choice tasks

Before analyzing the pictures in our database, we wanted to check whether the number of Instagram Likes is actually related to aesthetic preference and it is thus reasonable to use Likes as a proxy for aesthetic appeal. We asked independent participants for their aesthetic preferences for a subset of photographs from the database. In a second step, we tested to what extent formal balance measures (computation is explained in the Method section of Experiment 2) reflect perceptual balance by asking participants to judge the balance of photographs.

### Method

We recruited 30 participants via PsyKonLabs (ORSEE, Greiner, 2015), an online recruitment system for psychological experiments at the University of Konstanz, and asked them to perform an online survey that lasted about 20 min and was remunerated with a 3€ voucher. The participants received a link to the survey and used their own device. We asked them not to use tablets or smartphones and gave them instructions on how to calibrate their screen: They were instructed to set brightness on maximum and switch to full screen mode by pressing F11. Participants were also instructed to adjust the size of a standard page by using “CTRL +” or “CRTL –” to zoom in or out, in order to see the whole page maximally sized without the need to scroll down. Participants came from different academic disciplines (Psychology, Law, Politics, Economics, Biological Science, Sociology, and Architecture). Their average age was 25 years (*SD* = 8.65, age ranging from 18–52) and 7 of them were males and 23 females. The experiment was performed in accordance with the ethical standards laid down in the 1964 Declaration of Helsinki and its later amendments. In agreement with the ethics and safety guidelines at the Universität Konstanz, we obtained an informed consent statement from all individuals. They were informed of their right to abstain from participation in the study at any time without reprisal.

In the experiment, 96 photographs from our database were used (Table 2); the 12 most and the 12 least liked photographs of Maik Lipp (*M* = 2572, *SD* = 402 vs. *M* = 647, *SD* = 56.3) and Matthieu Venot (*M* = 1384, *SD* = 108 vs. *M* = 803, *SD* = 94.1), based on the number of Instagram Likes. In addition, we used the 12 most and 12 least balanced photographs of Sebastian Weiss (*M* = 1.31, *SD* = 0.35 vs. *M* = 24.7, *SD* = 3.61) and Kai Ziehl (*M* = 2.58, *SD* = 1.23 vs. *M* = 24.3, *SD* = 3.81), according to the computed balance scores (see below).

**Table 2:** Descriptive statistics for the photographs used in study 1.

	**Maik Lipp**			**Matthieu Venot**			**Sebastian Weiss**			**Kai Ziehl**		
	***N***	***M***	***SD***	***N***	***M***	***SD***	***N***	***M***	***SD***	***N***	***M***	***SD***
Most Instagram Likes	12	2,572	402	12	1,384	108						
Least Instagram Likes	12	647	56.3	12	803	94.1						
Best Balance Scores							12	1.31	0.346	12	2.58	1.23
Worst Balance Scores							12	24.7	3.61	12	24.3	3.81

*As balance measure, we used the Bal_DCM_ scores, introduced in the methods section of experiment 2*.

**Figure 4:**
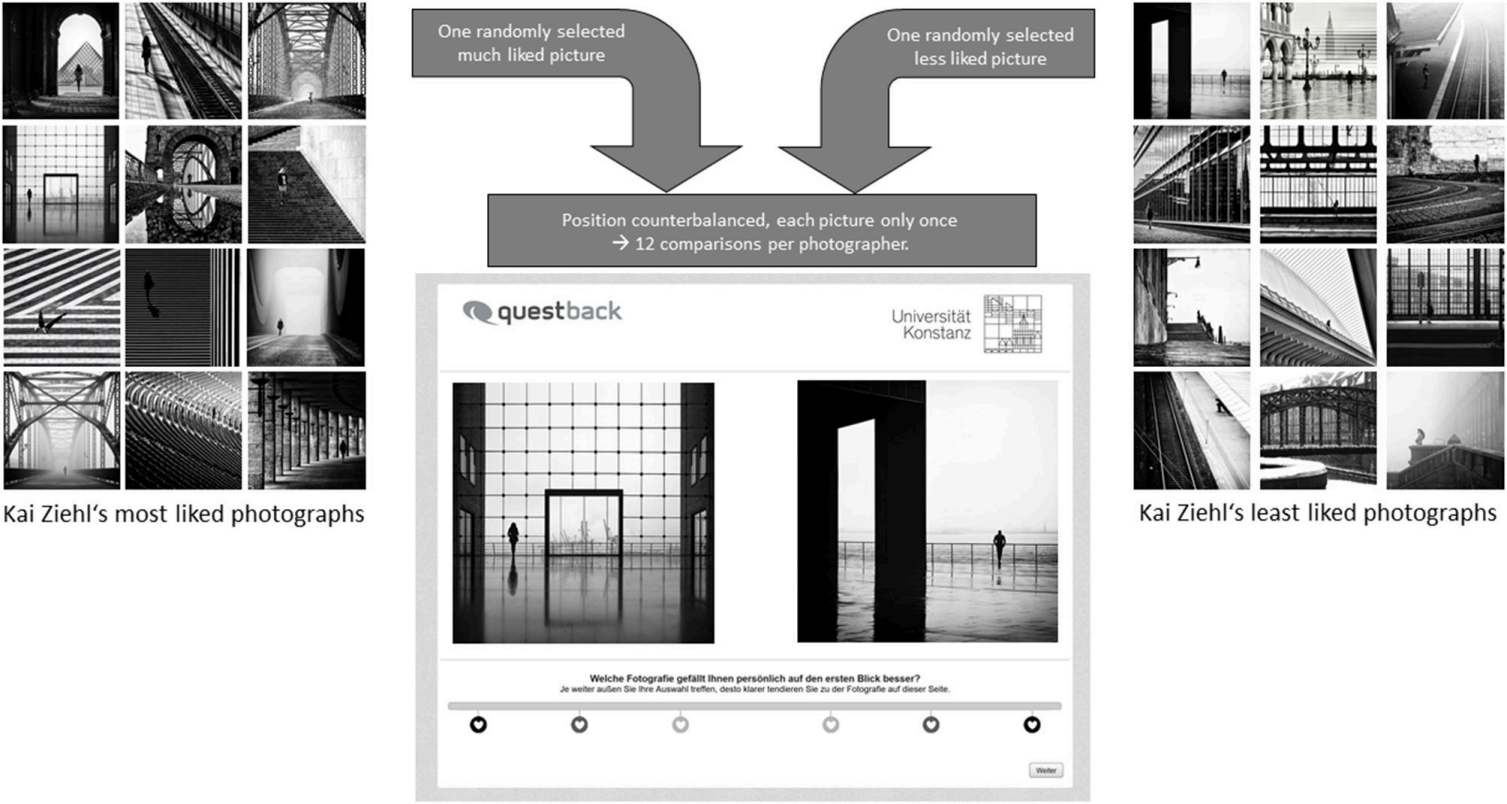
Experimental design explained with the example of Kai Ziehl's most and least liked photographs on Instagram. Participants had to make 12 decisions per photographer. We used images of two photographers classified by the number of Instagram Likes and of two additional photographers sorted by balance scores. This resulted in 24 Liking and 24 Balance judgments per participant. Each picture had an extension of 640 × 640 pixels. The original German question was “Welche Fotografie gefällt Ihnen persönlich auf den ersten Blick besser?” (engl. Which photo do you like better at first glance?) and “Welche Bildkomposition ist Ihrer Einschätzung nach in Bezug auf Helligkeit besser ausbalanciert?” (engl. Which photograph has a more balanced composition in respect to its distribution of brightness?) respectively.

In total, every participant performed 48 two-alternative forced choices (2AFCs), in which one of the 12 most liked photographs was randomly paired with one of the 12 least liked photographs of the same photographer. The same was done for balance. Thus, participants first judged 24 pairs in terms of liking (2AFC_Liking_) and afterwards another 24 pairs in terms of balance (2AFC_Balance_). Every participant viewed each of the 96 images only once to eliminate effects of repeated stimulus presentation. Thus, balance judgments were given for a different set of photos than liking judgments. The positions (left or right) of the “good” and “bad” photographs were counterbalanced and randomized across photographs and participants to control for position effects. It should be noted that the least liked and least balanced images were not recognizably of poor photographic quality, as the whole database consists of published work of professional photographers posting exclusively high-quality content. Consequently, the difference between “good” and “bad” photographs is not obvious at first glance. Figure 4 shows the setup of the experiment using the example of Kai Ziehl's most and least liked photographs.

**Figure A1:**
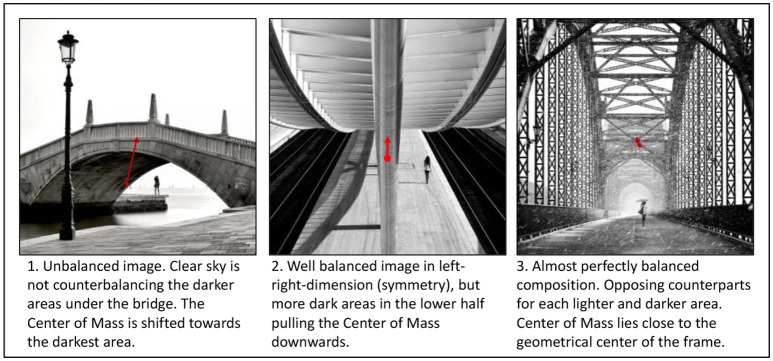
The red dot indicates the objective luminance Center of Mass, according to the DCM measure. The arrow is pointing towards the geometric center of the composition. The shorter the arrow, the better balanced the composition. DCM scores are as follows: Photo 1. BAL_DCM_ = 27.4; Photo 2. BAL_DCM_ = 5.27; Photo 3. BAL_DCM_ = 1.54.

The 2AFC_Liking_ task was based on the question “Which of these two photographs do you like better at first glance?.” The answer was given on a six-point Likert scale, so that participants not only chose one of the two images, but also weighted their judgment to indicate how strongly they preferred one image over the other. We used this as a confidence measure: Ratings on the outer edges of the scale indicate very confident decisions, whereas ratings close to the midpoint (which was not selectable) indicate rather indecisive choices. Figure 4 illustrates the display screen of the task. The subsequent 2AFC_Balance_ task used an analogous method and was based on the question “Which photograph has a more balanced composition in respect to its distribution of brightness?.” As the balance score measures luminance balance (see below), we presented grayscale versions of the original photographs. Thus, the participants saw the exact same image that was used for calculating balance scores. The concept of luminance balance was explained to the participants based on three examples of more vs. less balanced photographs. You find these examples in **Figure A1**.

### Results

For a first analysis, the responses on the six-point Likert scale were treated as binary, i.e., as preference for one of the stimuli in a pair. Every single decision could either match or mismatch the suggestion of the underlying numbers of Instagram Likes and balance scores, respectively. Confidence, as indicated by the exact value on the six-point scale, is considered below. If people chose their preferred images randomly, we would find 50% matching and 50% mismatching decisions. We analyzed the data both on task and participant level. On task level, we had a total of 720 decisions for balance and liking each. Our data revealed that 65% of the liking decisions matched the predictions based on Instagram Likes. Likewise, 64% of the balance decisions matched the prediction of the balance measure (see Table 3). The relation gets even more pronounced when less confident decisions—responses on the two midpoints of the Likert scale—are excluded. An analysis of the remaining “confident” decisions (*n* = 472) revealed 69% accordance with the prediction for liking decisions, and 68% accordance with the prediction for balance decisions. If we only consider “highly confident” choices, where only the outer extremes are analyzed (*n* = 168), matches increase to 71 and 77% respectively. Binomial tests proved all of these proportions to be significantly above chance levels (*p* < 0.001, two-tailed) in line with our predictions. Table 3 sums up these findings.

**Table 3:** Absolute case numbers and percentages of matches that support the predictions of Instagram Likes and balance scores, respectively.

	**Liking 2AFC task**			**Balance 2AFC task**		
	***N***	**Matches**		***N***	**Matches**	
All decisions	720	468	65%	720	459	64%
“Confident” decisions (2 midpoints excluded)	472	326	69%	510	345	68%
“Highly confident” decisions (4 midpoints excluded)	168	120	71%	137	106	77%

To check whether the results were only true for some participants, we also looked at each participant's decisions separately. Each participant made a total of 24 liking and 24 balance decisions. We created two binomial variables 2AFC_Liking_ and 2AFC_Balance_, where 1 indicates decisions in accordance with numbers of Instagram Likes or balance scores. For each participant (*N* = 30) mean matching scores were calculated, where values above 0.5 indicate a higher number of matches than mismatches, 0.5 representing random decisions. The mean 2AFC scores per participant ranged from 0.33 to 0.83 for liking (*M* = 0.65, *SD* = 0.11) and from 0.25 to 0.92 (*M* = 0.64, *SD* = 0.19) for balance. On a descriptive level, 28 out of 30 participants preferred images with more Likes more often, in line with our prediction. Likewise, 24 out of 30 participants chose images with better balance scores more often, when asked for balance. A single sample *t*-test was conducted to determine whether participant's means are significantly higher than chance probability (μ_0_ = 0.5) for both balance and liking. Table 4 shows that participants were significantly more likely to choose the photograph with the higher number of Instagram Likes, when asked for liking preferences, *t*_(29)_ = 7.64, *p* < 0.001, *Cohen's d*^2^ = 1.36. Likewise participants were more likely to select the objectively more balanced photograph, when enquired about good balance, *t*_(29)_ = 4.00, *p* < 0.001, *Cohen's d* = 0.72.

**Table 4:** One-sample *t*-test for participant means for the Liking and Balance 2AFC tasks.

	**Test value** = **0.5**						
	***t***	**df**	***p***	**Mean Difference**	**95% Conf. Interval**		***Cohen's d***
					**Lower**	**Upper**	
2AFC Liking	7.64	29	<0.0001	0.15	0.11	0.19	1.36
2AFC balance	4.00	29	<0.0001	0.14	0.068	0.21	0.72

### Discussion

The results of the 2AFC task for liking show that aesthetic preferences can be predicted, at least to some extent, by the number of Instagram Likes. A large-sized *Cohen's d* suggests that individuals under laboratory conditions more often prefer the one of two photographs that received more Likes on Instagram, when asked for aesthetic preference. In our particular experimental design with architectural photographs, the aesthetic appeal of a picture seems to be reflected to a certain extent by liking activity of the Instagram community. This was the case even though we asked participants for their spontaneous personal preferences, instead of an assessment of general aesthetic appeal. It remains open whether the relation would have been stronger if we had ask participants for general judgments of aesthetic appeal, as research suggests (cf. Hager et al., 2012). Clearly, more research is needed to understand the nature of Instagram Likes and their connection with the aesthetic appeal of images. Our approach is a promising first step.

Concerning balance, the participants' assessments mostly matched the predictions made with the computed balance measure. A medium effect size supports the hypothesis that quantitative balance measures validly relate to subjectively assessed perceptual balance. The fact that all photographs are of high quality and participants had to choose between pairs of photographs taken by the same photographer makes these findings even more persuasive. Likewise, the more confident the participants were in their choice, the more often it matched the quantitative balance measure. However, it must be noted that gender was unbalanced (23 female to 7 male) in our sample. Aesthetic preference has been shown to differ between females and males (e.g., Cela-Conde et al., 2009). Our results are also limited to our specific stimulus set of minimalistic photographs of architecture.

Despite these limitations, our results provide evidence for both the conception of Instagram Likes as a proxy for aesthetic appeal and quantitative balance scores as a reliable measure of perceptual balance. These findings provide the basis for further analyses of the relation between Instagram Likes and objective balance measures of image composition.

## Experiment 2: analysis of our Instagram database

In this part of our study, we analyzed to what extent formal measures of visual balance and curvature can account for Instagram Likes. In the following, we first describe how the Instagram liking data and the images were prepared, and how the objective measures of visual balance were computed. In addition to the Instagram photos, we also calculated balance measures for a set of 52 randomly shot control photographs. By analyzing these data, we hoped to gain a deeper understanding of the relationship between balance and aesthetic appeal. We also examined differences between compositions with “2D” vs. “3D” appearance that differ in complexity.

### Method

#### Pre-processing

On Instagram, the number of Likes is strongly influenced by the different sizes of followerships of different accounts. To control for this confound, the absolute numbers of Likes were z-standardized per photographer. Thus, we were able to compare relative amounts of Likes between different accounts.

The photographs were processed and analyzed with Matlab R2012b (The Mathworks, Natick, MA, USA). First, all images were resized to 640 × 640 pixels. Because the balance measures are based on the distribution of luminance and therefore the gray level of the pixels, images were converted with Matlab's *rgb2gray*^3^. In line with previous research, it is assumed that dark areas are perceptually heavier than bright areas (e.g., McManus et al., 2011; Hübner and Fillinger, 2016). As each pixel in a standard 8-bit-deep image has a gray level ranging from 0 for black pixels to 255 for white pixels, we used negatives of the images to reverse the scale for our calculations.

**Figure A2:**
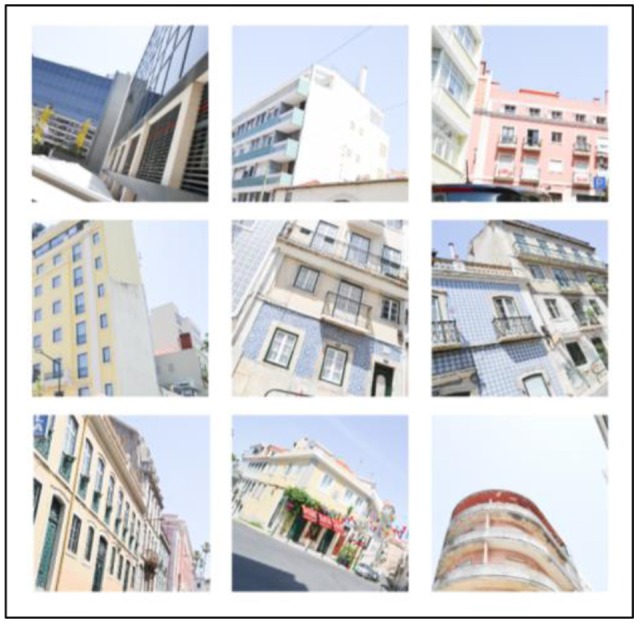
Some examples of the randomly shot photographs from study 2.

Our randomly shot control photographs were taken “from the hip” without even looking through the viewfinder or attempting to keep the camera steady. One of the authors (KT) shot the photos with a Nikon DSLR in high quality and subsequently cropped them to quadratic format. You find some examples of the randomly shot images in Figure A2 (Appendix). The photos were preprocessed as the other pictures.

#### Two measures for symmetry

In his *Theory of Pure Design* Denman Ross states: “In Symmetry we have a balance which is perfectly obvious and instinctively felt by everybody.” (Ross, 1907, p. 20). When we think of symmetry as a perceptual feature, the most obvious symmetry is bilateral symmetry around a vertical axis, also called left-right symmetry. According to Osborne, “[s]ymmetry is also sometimes […] a quality of the composition as a whole when there is no exact duplication of forms but a certain balance, or equality of ‘weighting,’ about an imaginary axis.” (Osborne, 1986, pp. 80–81). Hence, Osborne conceptualized symmetry as an aspect of balance. In empirical research, Hübner and Fillinger (2016) have reported strong correlations between their balance measures and subjective symmetry ratings. Their results suggest that balance along the vertical axis (left-right balance) is most strongly connected with symmetry. As a consequence, we decided to use left-right balance as a measure of symmetry and utilized two different measures that have repeatedly been used in previous research. The first calculates mass proportions along different axes of the image (Wilson and Chatterjee, 2005; Hübner and Fillinger, 2016), the second computes the center of mass and measures its distance to the geometrical center of the frame (Bauerly and Liu, 2006; McManus et al., 2011; Hübner and Fillinger, 2016). For both measures it is possible to look at the left-right dimension separately, which we use as measures of symmetry. Note that this is not mirror symmetry, but a measure of lateral balance around the vertical axis in the sense of Osborne (1986).

**Figure 5:**
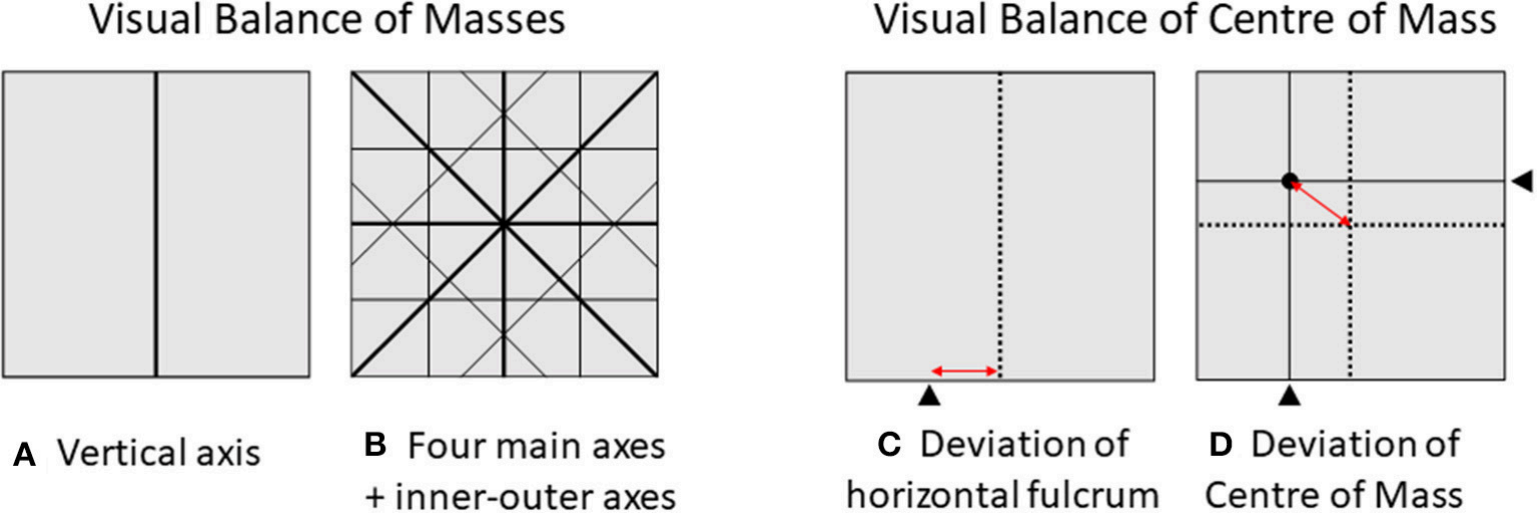
These examples illustrate the calculation of the visual balance measures. **(A)** Balance along the vertical axis is the Symmetry *SYM*_*M*_. **(B)** The four main balance axes and their corresponding inner-outer dimensions for the calculation of *BAL*_*M*_. The eight axes are: vertical (verti), horizontal (hori), diagonal left top to right bottom (dia1), diagonal left bottom to right top (dia2), and their inner-outer counterparts (ioverti, iohori, iodia1, iodia2). **(C)** Horizontal center of mass and distance to geometrical center for calculation of *SYM*_*DCM*_. **(D)** Black fulcrums indicate the horizontal and vertical center of mass (*CM*_*hori*_ *and CM*_*verti*_), the black dot indicates the center of mass, and the red arrow indicates the Euclidian distance of the center of mass from the geometrical midpoint of the frame. For an example of the center of mass and the resulting *BAL*_*DCM*_ scores in photographs from the Instagram database see Figure A1 in the Appendix.

For the first measure, assume that an image is divided along the vertical axis into two equally sized rectangles *I*_*left*_ and *I*_*right*_ (see Figure 5A). Based on Wilson and Chatterjee's balance score—their so-called APB (Wilson and Chatterjee, 2005)—the algorithm adds up all pixels' luminance values for the whole image *I*_*ij*_ and its halves *I*_*left*_ and *I*_*right*_. The masses *M* are calculated as

(1) $$\begin{array}{r} M_{left}=\overset{\frac{w}{2}}{\underset{i=1}{\sum }}m_{i}, \end{array}$$

(2) $$\begin{array}{r} M_{right}=\;\overset{w}{\underset{i=\frac{w}{2}}{\sum }}m_{i}, \end{array}$$

(3) $$\begin{array}{r} M_{image}=\;\overset{w}{\underset{i=1}{\sum }}m_{i}, \end{array}$$

where *w* is the width of the image and *m*_*i*_ is the mass sum score of all pixel luminance values in column *i*. The difference between the masses *M*_*left*_ and *M*_*right*_ is then divided by the sum of all pixels' masses *M*_*image*_ and multiplied by 100, which makes the resulting symmetry score range from 0 to 100. As a result, smaller scores indicate more symmetry. If *M*_*left*_ and *M*_*right*_ are equal, symmetry of masses *SYM*_*M*_ is 0 and therefore maximal.

(4) $$\begin{array}{r} SYM_{M}=\;\frac{\left|M_{left}\;M_{right}\right|}{M_{image}}*100 \end{array}$$

You can visualize this symmetry score *SYM*_*M*_ by thinking of weighing scales, where you put the two halves of the photograph on both sides. Weight in this case is visual mass for which we use the pixels' luminance values. However, this measure does not take the distance of masses to the vertical axis into account.

Thinking of the childhood experience of seesawing, one might object that it does not necessarily require a partner of exactly your weight—position matters also. Therefore, we computed a second symmetry measure in analogy to physics following the concept of the Center of Mass (*CM*) used by Bauerly and Liu (2006) and developed further by McManus et al. (2011) and Hübner and Fillinger (2016). It measures the deviation of the center of mass (*DCM*) from the geometrical center of an image (see Figure 5C). Equivalent to *SYM*_*M*_, we computed the deviation of the horizontal center of mass from the vertical midline as a second measure for symmetry. First, we calculated the horizontal center of mass as the distances of masses from an arbitrary reference point, for which we chose the left end of the image (position *x* = *0*) (see McManus et al., 2011, p. 618 for a detailed description). In the pixel matrix *I*_*ij*_ we calculated the center of mass for the horizontal dimension as

(5) $$\begin{array}{r} CM_{hori}=\;\frac{\sum_{i=1}^{w}m_{i}+r_{i}}{\sum_{i=1}^{w}m_{i}}, \end{array}$$

where *w* is the image's width, *m*_*i*_ is the sum score of all pixels' masses in column *i* and *r*_*i*_ is the distance of column *i* from the reference point at the left side of the image. *CM*_*hori*_ is illustrated in Figure 5A as a fulcrum beneath the image. We normalized *CM*_*hori*_ by dividing by *w*, resulting in ${CM^{\prime }}_{hori}$, ranging from 0 to 1:

(6) $$\begin{array}{r} {CM^{\prime }}_{hori}=\frac{CM_{hori}}{w}. \end{array}$$

${CM^{\prime }}_{hori}$ coincides with the geometrical midline, if it has the value 0.5. Therefore, the horizontal distance between the center of mass and the geometrical center is

(7) $$\begin{array}{r} d_{CM_{hori}}=0.5-{CM^{\prime }}_{hori}. \end{array}$$

As a second symmetry measure we then used the relative *DCM* from the center of the frame in percent. Again, lower scores mean more symmetry.

(8) $$\begin{array}{r} SYM_{DCM}=\frac{\sqrt{d_{CM_{hori}}^{2}}}{0.5}\;*\;100. \end{array}$$

#### Two measures for overall balance

The described lateral balance measures can also be extended to a more general balance measure by incorporating more axes.

First, we explain the “weighting scale” balance measure based on Wilson and Chatterjee (2005) that is an extension of *SYM*_*M*_. The calculation follows the same logic as formula (1) to (4) for seven more axes, which can be seen in Figure 5B. *SYM*_*M*_ is now called *verti*_*M*_, as it compares masses around the vertical axis. The resulting balance score of masses is the mean of all eight partial measures, ranging from 0 to 100:

(9) $$\begin{array}{r} BAL_{M}=\frac{\begin{array}{c} verti_{M}+hori_{M}+{dia1}_{M}+{dia2}_{M}+ioverti_{M}\\ \;+\;iohori_{M}+{iodia1}_{M}+{iodia2}_{M} \end{array}}{8}. \end{array}$$

Second, we describe the “seesaw” balance measure based on McManus et al. (2011) that is a continuation of *SYM*_*DCM*_. The distance of the vertical fulcrum from the vertical midline is calculated analogously to the horizontal dimension (see Figure 5D). The center of mass for the whole composition then lies on the intersection point of vertical and horizontal center of mass (see Figure 5D). The deviation measure of balance is defined by the Euclidean distance of the two-dimensional center of visual mass to the geometrical center of the image (indicated by the red arrow in Figure 5D). The resulting balance score is the relative deviation in percent, where again, low values mean more balance, in terms of the center of mass being located closer to the midpoint:

(10) $$\begin{array}{r} BAL_{DCM}=\frac{\sqrt{d_{CM_{verti}}^{2}+d_{CM_{hori}}^{2}}}{\sqrt{0,5}}\;*\;100. \end{array}$$

#### Classification of curvature and angularity

**Figure 6:**
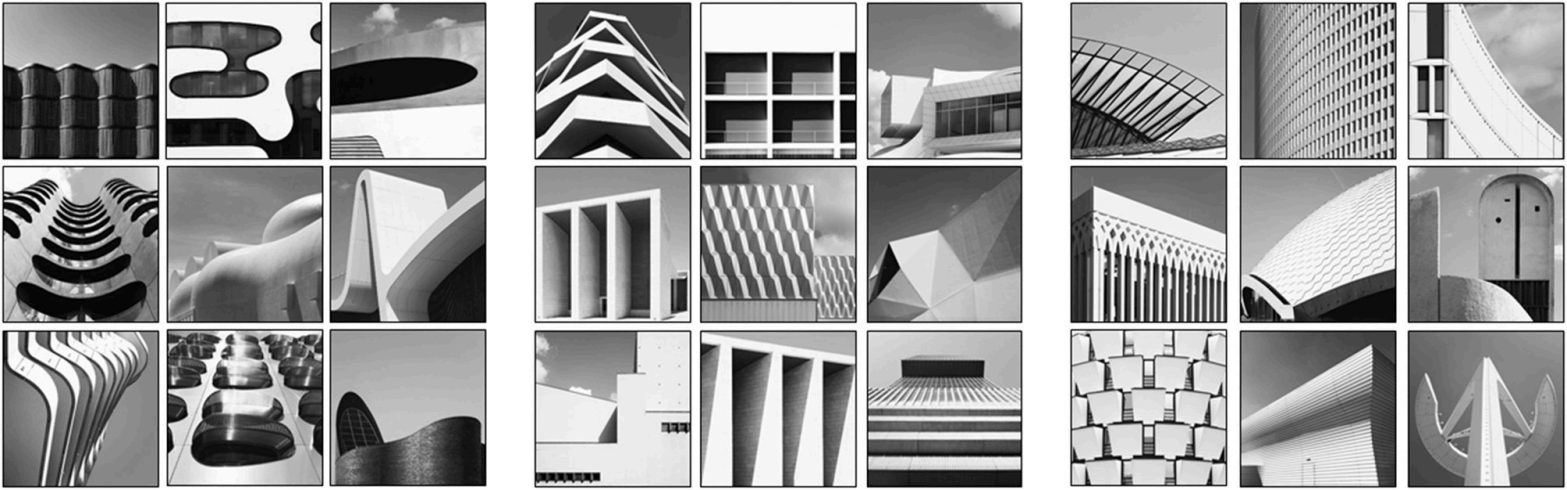
From left to right: Nine examples of curved, angular, and mixed compositions in photographs of Sebastian Weiss.

One of the authors (KT) and two independent persons classified all images in our database as being either curved, angular, or mixed. Only when all three judges agreed, photographs were classified as either curved or angular. Ambiguous classifications were sorted in the mixed category. This final classification over agreement of three judges was only done for one of the photographers (SW, *N* = 268), because the sample size of curved compositions was too small for the other photographers. Figure 6 shows examples for clearly curved (*n* = 44) and angular (*n* = 80) compositions from Sebastian Weiss.

### Results

We first present descriptive statistics of balance measures and liking data separately for each photographer. For comparison, we also report visual balance measures for a set of randomly shot photos. We then report the results of a linear regression analysis of the visual balance measures to predict Instagram Likes, for “2D” and “3D” compositions respectively. Finally, we present the result with respect to curvature.

#### Descriptive statistics of visual balance measures

We calculated measures for symmetry (*SYM*_*M*_ and *SYM*_*DCM*_) and balance (*BAL*_*M*_ and *BAL*_*DCM*_) for all photos in our database, and for a control set of 52 randomly shot photographs. Table 5 shows descriptive statistics for all five measures per photographer, as well as significant differences between photographers, which exist in some cases. Alex Hamburg's photographs are more symmetric and also generally more balanced than the images of the other photographers and the random photos. The randomly shot control photographs score higher than Alex Hamburg's and Sebastian Weiss' photos in all balance measures and are thus less balanced. They are less symmetric than photographs by Maik Lipp and less balanced than images by Matthieu Venot and Kai Ziehl.

**Table 5:** Means and standard deviations for all measures.

	**Alex Hamburg**		**Maik Lipp**		**Matthieu Venot**		**Sebastian Weiss**		**Kai Ziehl**		**Random Photos**	
	***N*** = **244**		***N*** = **49**		***N*** = **61**		***N*** = **268**		***N*** = **57**		***N*** = **52**	
	***M***	***SD***	***M***	***SD***	***M***	***SD***	***M***	***SD***	***M***	***SD***	***M***	***SD***
SYM_M_	5.34^a^	8.03	10.1	12.2	14.1	15.6	11.8	10.9	14.6	15.5	17.3^b^	15.4
SYM_DCM_	3.15^a^	4.84	6.19	6.64	8.19	9.10	7.06	6.66	8.78	9.62	10.1^b^	9.10
BAL_M_	5.21^a^	5.01	13.4	7.27	13.0	7.56	11.6	5.89	13.1	6.62	15.5^b^	5.68
BAL_DCM_	4.39^a^	5.23	11.8	7.08	11.8	8.74	9.60	5.88	11.2	8.18	14.3^b^	7.52

*Index numbers indicate significant differences between photographers based on two-sided tests assuming equal variances. Significance level is p < 0.05. Tests are adjusted for all pairwise comparisons within a row of each innermost sub-table using the Bonferroni correction*.

a *Values are significantly lower (and therefore more balanced) than in all other photographers*.

b *Values for random photographs are significantly higher (and therefore less balanced) than AH and SW in all measures; significantly higher than ML in Sym_M_ and Sym_DCM_; significantly higher than MV in Bal_M_; and significantly higher than KZ in Bal_M_ and Bal_DCM_*.

Overall, the visual balance measures are strongly intercorrelated as Table 6 shows. The two different versions of symmetry and balance scores correlate with *r* = 0.95 and *r* = 0.91, respectively. Correlations between symmetry and balance scores range from *r* = 0.57 to *r* = 0.62.

**Table 6:** Intercorrelations between the different measures of visual balance for all 679 photographs.

	**Pearson correlations**			
	**SYM**_M_	**SYM**_DCM_	**BAL**_M_	**BAL**_DCM_
SYM_M_	1	0.95^**^	0.57^**^	0.58^**^
SYM_DCM_		1	0.58^**^	0.61^**^
BAL_M_			1	0.91^**^
BAL_DCM_				1

P-values for Pearson correlations ^*^p < 0.05,

** *p < 0.01*.

#### Linear regression analysis of visual balance measures and Instagram likes

To examine the effects of balance on aesthetic appeal, we first computed Pearson's correlation coefficients and corresponding *R*^2^. Note that smaller values of visual balance measures indicate more balance. Therefore, negative correlations indicate a positive relationship between balance and the number of Likes. Our analyses revealed that the more symmetric and balanced a photo is, the more Likes it received on Instagram. As Table 7 shows, there were negative correlations between all visual balance measures and Likes for all photographers, except Alex Hamburg.

**Table 7:** Correlation analysis per photographer, Pearson correlation coefficients, and *R*^2^.

		**SYM_M_**	**SYM_DCM_**	**BAL_M_**	**BAL_DCM_**	**Time effects**
Alex Hamburg, “2D” *N* = 132	Correlation with Likes	0.29^**^	0.27^**^	0.15	0.26^**^	0.008
	*P*	0.001	0.002	0.097	0.002	0.93
	*R*^2^	0.084^**^	0.075^**^	0.021	0.070^**^	
Alex Hamburg, semi “2D” *N* = 112	Correlation with Likes	0.061	0.005	−0.057	−0.10	−0.18
	*P*	0.52	0.96	0.55	0.28	0.063
	*R*^2^	0.004	0.000	0.003	0.011	
Maik Lipp *N* = 49	Correlation with Likes	−0.11	−0.15	−0.36^*^	−0.37^**^	0.083
	*P*	0.456	0.31	0.011	0.009	0.57
	*R*^2^	0.012	0.022	0.13^*^	0.14^**^	
Matthieu Venot *N* = 61	Correlation with Likes	−0.20	−0.23	−0.37^**^	−0.30^*^	−0.027
	*P*	0.13	0.076	0.004	0.018	0.84
	*R*^2^	0.04	0.053	0.14^**^	0.092^*^	
Sebastian Weiss (controlled for time effects) *N* = 268	Correlation with Likes (partial correlations)	−0.11 (−0.10)	−0.11 (−0.094)	−0.29** (−0.28)	−0.25** (−0.21)	0.44^**^
	*P*	0.076 (0.098)	0.079 (0.125)	0.000 (0.000)	0.000 (0.000)	0.000
	*R*^2^	0.012	0.012	0.08^**^	0.060^**^	
Kai Ziehl *N* = 57	Correlation with Likes	−0.25	−0.20	−0.37^**^	−0.33^*^	−0.23
	*P*	0.06	0.13	0.005	0.012	0.082
	*R*^2^	0.06	0.041	0.14^**^	0.11^*^	
Photographs with “2D” appearance *N* = 132	Correlation with Likes	0.29^**^	0.27^*^	0.15	0.26^**^	
	*P*	0.001	0.002	0.097	0.002	
	*R*^2^	0.084^**^	0.075^**^	0.021	0.070^**^	
Photographs with “3D” appearance *N* = 435	Corr. with Likes (z–Scores)	−0.14^**^	−0.15^**^	−0.32^**^	−0.28^**^	
	*P*	0.003	0.002	<0.0001	<0.0001	
	*R*^2^	0.021^**^	0.021^**^	0.10^**^	0.077^**^	

Negative correlations indicate that more visual balance accounts for more Instagram Likes. Time effects using the chronological order of upload time. P-values for correlations

* *p < 0.05*,

** *p < 0.01*.

In the following, we refer to Maik Lipp, Matthieu Venot, Sebastian Weiss, and Kai Ziehl as “3D” photographers. For Alex Hamburg's “2D” photographs, balance correlates positively with Instagram Likes, whereas for his “3D” images balance is not related to the number of Instagram Likes. The **symmetry measures** *SYM*_*M*_ and *SYM*_*DCM*_ were correlated with the Likes only for Alex Hamburg's “2D” photos (*r* = 0.29 and *r* = 0.27), indicating that more symmetric photographs were liked more. For all “3D” photographers, correlations with symmetry tend to be negative without reaching significance. The **balance measures** *BAL*_*M*_ and *BAL*_*DCM*_ correlate negatively with Likes for “3D” photographers, ranging from *r* = −0.21 to *r* = −0.45. For Alex Hamburg's “2D” photographs one of the balance scores correlates significantly positive (*r* = 0.26) with Likes. To control for possible time effects on the number of Instagram Likes, we also generated a time variable by numbering the photographs in chronological order. The last column in Table 7 shows the corresponding effects. Only for Sebastian Weiss there is a significantly positive correlation, meaning that images posted later during our collection time frame (ranging from February 2014 to November 2016) received more Likes. The time frames for all photographers are given in the description beneath Table 1. Importantly, controlling for this effect did not change the relation between balance and Instagram Likes, as you can see in Table 7 (data in parentheses in the Sebastian Weiss row).

**Figure 7:**
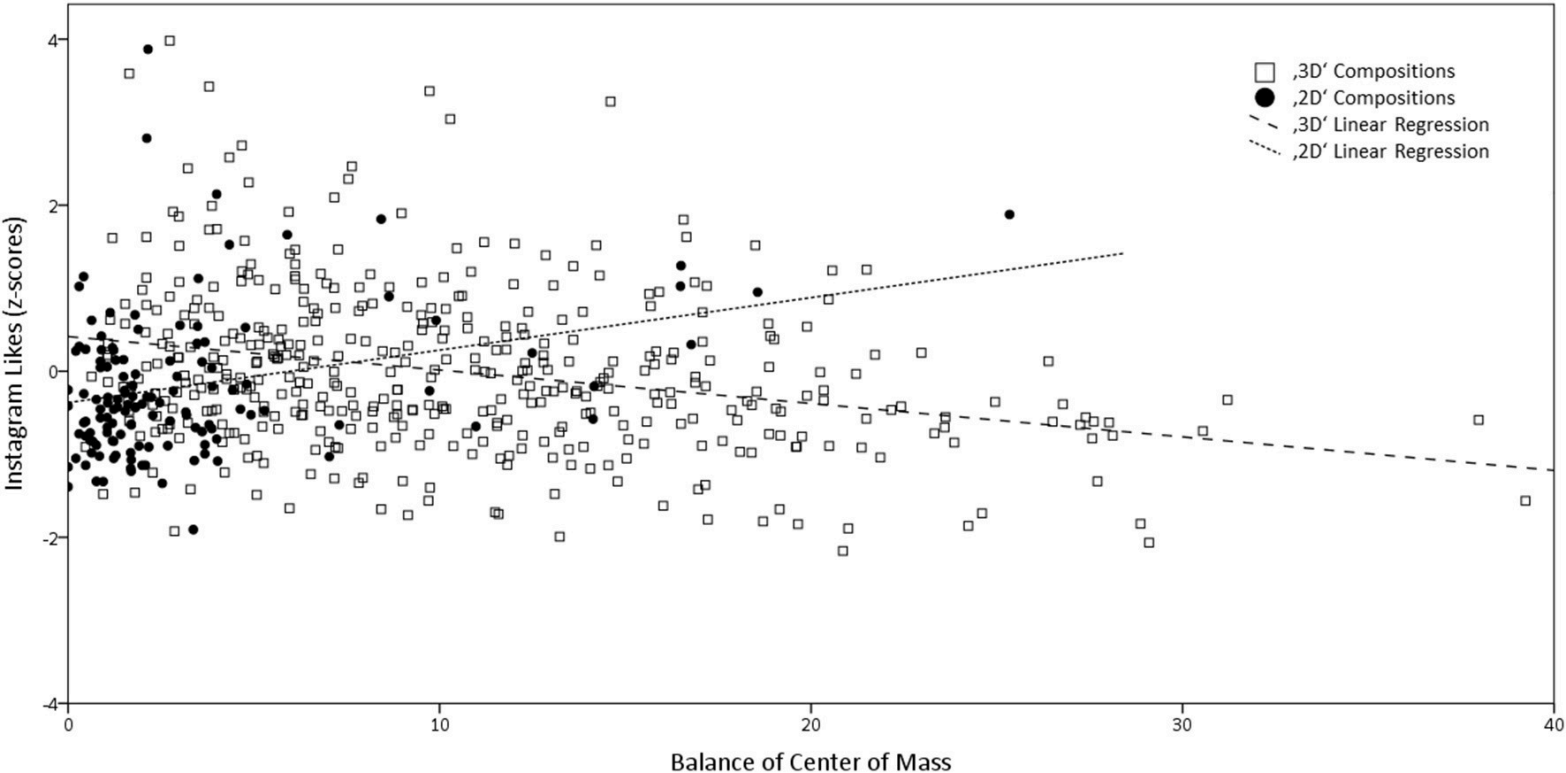
This figure illustrates the difference between “2D” vs. “3D” photographs in visual balance effects on Instagram *Likes*, using the example of the *BAL*_*DCM*_ measure. Depicted are “2D” compositions by Alex Hamburg (*n* = 132, *r* = 0.26**) vs. “3D” compositions by the other four photographers (*n* = 435, *r* = −0.26**). *P*-values for Pearson correlations *p < 0.05, **p < 0.01.

In Alex Hamburg's **“2D” photographs**, for both symmetry measures significant regression equations were found [*F*_(1, 130)_ = 11.9, *p* < 0.001 and *F*_(1, 130)_ = 10.5, *p* = 0.002], with *R*^2^ of 0.084 and 0.075 respectively. The location of the center of mass relative to the geometrical center (BAL_DCM_) also explains 7% of variance [*F*_(1, 130)_ = 9.74, *p* = 0.002], whereas BAL_M_ does not explain variance. For none of the photographers of **“3D” photographs**, symmetry explains any variance. But for all of them, balance measures BAL_M_ and BAL_DCM_ generated significant regression equations, with *R*^2^ ranging from 0.060 to 0.14. Taken together, the most conclusive visual balance measures for all “3D” photographs are BAL_M_ [*F*_(1, 433)_ = 48.7, *p* < 0.001], with an *R*^2^ of 0.10. Figure 7 illustrates the difference in balance effects in “2D” vs. “3D” compositions. Multilinear regression analyses with more than one of the visual balance measures were not found to significantly increase the model's accuracy.

#### Effects of curvature

For investigating the effect of curvature, we analyzed Sebastian Weiss' photographs (*n* = 268), because they included sufficient numbers of angular, curved, and mixed compositions (*n*_*curved*_ = 44, *n*_*angular*_ = 80, *n*_*mixed*_ = 144). As described above, the pictures were classified by three independent judges. Interrater reliability was estimated using the percentage of agreement across multiple judges (McHugh, 2012). For 67% of the photos all three judges agreed in their decisions, in nearly 33% two of the three agreed (disagreement always in question of either curved-mixed or angular-mixed), and there was only one case of discordant judgments (one judge rated the image as curved, one as angular, and one as mixed). Photographs thus were divided into three categories: curved compositions (*M*_*Likes*_ = 3,329, *SD* = 623), angular compositions (*M*_*Likes*_ = 3073, *SD* = 666), and mixed compositions (*M*_*Likes*_ = 3,033, *SD* = 678). Homogeneity of variances was asserted using Levene's Test which showed that equal variances could be assumed (*p* = 0.61). Instagram Likes were normally distributed for mixed compositions, but not for curved and angular compositions, as assessed by the Shapiro-Wilk test (α = 0.05). A one-sided one-way ANOVA revealed that the aesthetic appeal of the photographs (as measured by the number of Instagram Likes) differed significantly for the different categories of curvature, *F*_(2, 265)_ = 3.38, *p* = 0.036, η^2^ = 0.025. Planned additional tests showed that this significant difference was a preference for curved compositions over angular compositions [*t*_(122)_ = 2.09, *p* = 0.039, *Cohens's d* = 0.40] and mixed compositions [*t*_(186)_ = 2.58, *p* = 0.011, *Cohens's d* = 0.45]. There was no significant difference between angular and mixed compositions.

### Discussion

#### Effects of visual balance

By computing visual balance measures for photos published by different photographers on Instagram, we found several interesting results. First, “2D” photographs (by Alex Hamburg) are generally more balanced than “3D” photographs and show only little variance in visual balance measures. The considered “3D” photographs showed a similar balance pattern for all four “3D” photographers, as Table 5 shows. In comparison, randomly shot control photographs score, on average, higher in all balance measures and are thus less balanced than professional photographs. However, the differences are not very pronounced and not always significant. It is noteworthy that not even randomly shot pictures score on the extreme end of the theoretically possible range of 0–100. The maxima of the visual balance measures for the control photos range from 23.6 for *BAL*_*M*_ up to 71.5 for symmetry *SYM*_*M*_ (*SYM*_*DCM*_ = 41.9, *BAL*_*DCM*_ = 43.9).

The inter-correlation matrix for all visual balance measures revealed that the two versions of symmetry and balance measures—based on the ratios of masses (*SYM*_*M*_, *BAL*_*M*_) or the location of the center of mass (*SYM*_*DCM*_, *BAL*_*DCM*_) respectively—are highly correlated. The left-right dimensions of the two approaches that were used as symmetry measures seem to measure almost the same concept (*r* = 0.95), as do the two overall balance measures (*r* = 0.91). In contrast, overall balance measures correlate considerably less with symmetry measures (*r* ranging from 0.57 to 0.62) indicating that the concepts of symmetry and balance are distinguishable.

With respect to aesthetic liking, we found that for our architectural photographs with “3D” appearance, the visual balance measures account for 8–10% of the variance in Instagram Likes. More balanced pictures received more Likes. The opposite is true for “2D” photographs, where also 8% of variance could be explained, but more visual balance means less Likes. The best measure to predict Instagram Likes in “3D” photographs was *BAL*_*M*_. In “2D” pictures *SYM*_*M*_ performed best in predicting Likes. As Table 5 shows, *SYM*_*M*_ is also the visual balance measure with the most variance in “2D” photographs, all other measures cluster at the extreme end of maximum balance. Combining different measures did not improve prediction of Likes.

Taken together, we found consistent and reliable effects of balance on Instagram Likes over five different Instagram accounts, following two approaches to calculate balance measures. Our results show that the effect of balance on Likes depends on the level of balance in the pictures. Whereas for “3D” photographs, there is a positive relation between balance and Likes, the opposite holds for the “2D” pictures—the latter also had higher levels of balance. This might find explanation in an inverted U-shaped relation between balance and aesthetic appeal, similar to the relation proposed between complexity and liking (e.g., Berlyne, 1971; Imamoglu, 2000). It might hold true that more balance—as defined by formal measures—goes hand in hand with less complexity. A quadratic regression model with balance (*BAL*_*M*_) predicting Instagram Likes (z-scores) for all 679 images of our database, reveals 4% explained variance [y = 0.01 + 0.03x – 0.002x^2^; *F*_(2, 676)_ = 12.8, *p* < 0.001, *R*^2^ = 0.036], which is a numerically better fit than a linear regression model for the whole database [y = 0.24–0.02x; *F*_(1, 677)_ = 19.9, *p* < 0.001, *R*^2^ = 0.029]. This ad hoc result could be a promising starting point for further research.

#### Effects of curvature

With our selection of architectural photographs, we could replicate that curved compositions are preferred over angular and mixed ones (Bar and Neta, 2006; Munar et al., 2015). The result that angular compositions did not differ in Likes from our baseline of mixed images supports the idea that curvature has positive effects itself (Palumbo et al., 2015), which is not explained by an avoidance of sharp or angular objects, but with a special appeal implied in curved compositions. A potential limitation is that for architectural photographs curvature is possibly confounded with rarity and surprise, as architecture usually is of angular nature, due to practical reasons. On the other hand, a closer look reveals that in the investigated architectural photographs angularity is not generally uninteresting or boring (see Figure 6 for examples). Also, both curved and angular images in our database depict modern architecture of European metropolitan areas, therefore Zeitgeist factors (Carbon, 2010) should not play a role. The presence of the well-studied preference for curvature highlights the usefulness of Instagram Likes as a proxy for explicitly gathered liking ratings.

## General discussion

The current paper illustrates the potential of low-level features to predict aesthetic liking in real-life online data. Instagram Likes can be used in investigating the aesthetic appeal of visual stimuli. In Experiment 1 we have shown that if an architectural photograph has more Likes than another one, that was posted by the same photographer, it is on average also preferred in an experimental setting. In this sense, Instagram Likes can potentially be used as a proxy for the aesthetic appeal of a photograph. This finding is a promising starting point for different kinds of research projects based on freely accessible Instagram liking data. Clearly more research on the link between Likes and explicit rating or preference assessments is needed. However, our results give point to the hypothesis that using the numbers of Likes is an alternative to gathering liking data in surveys.

In Experiment 2, we examined our Instagram database in respect to visual features that are known to affect the aesthetic appeal of pictures. Specifically, we analyzed the relation between different measures of visual balance and the number of Likes. For pictures with a “3D” appearance, this relation was positive, whereas it was negative for photographs with a “2D” appearance. Because “2D” pictures were generally highly balanced, the overall results suggest that the relation between balance and aesthetic might have an inverted U-shape. Whether this is indeed the case, however, needs further investigation.

Our data also shows that curved photographic compositions receive more Instagram Likes than angular compositions. This supports the idea of a predominant preference for curvature in the visual domain (Munar et al., 2015; Palumbo et al., 2015). The curvature of the composition was assessed by subjective classification. However, it would be worthwhile to implement algorithms that objectively measure the degree of curved lines in a composition, to enable more objective investigation of the effects of curvedness on liking. Further research is needed to explore the relative importance of different compositional features.

Taken together, our study demonstrates that Instagram Likes for images on large accounts might be used as measure to investigate effects of objective image features on aesthetic appeal, at least for architectural photographs. For the examined measures of balance and curvature the effects were relatively small, i.e., explained about 6–14% of the variance of the Likes. However, one has to take into account that real photographs were used. In the studies of Wilson and Chatterjee (2005) and Hübner and Fillinger (2016), for instance, balance was manipulated experimentally and it was the only feature that varied across stimuli. Therefore, it is no wonder that the effects of balance were relatively large. Here, we merely restricted the pictures by using only architectural photographs in square format depicting minimalistic content. Nevertheless, there are still various features, such as color, contrast, content, etc. that vary across the pictures and that presumably affected the liking of the pictures (Arnheim, 1971). Moreover, Instagram Likes result from real-life data that is obviously influenced by a great number of confounding variables, such as the use of hashtags, the time of upload, tagged persons, and demographic factors of followers, which are difficult to control for. Given these various sources of uncontrolled variance, the observed effects of the considered features are remarkable, even if effect sizes are quite low. Moreover, our stimulus set is massively imbalanced with respect to quality, as only high-quality photographs have been used. Variance in aesthetic appeal would largely increase, if photographs of lower quality were also included, possibly leading to larger effect sizes. Bearing in mind the non-laboratory setting of this study, the reported small effect sizes suggest relatively robust underlying effects.

Restricting the types of photographs, which was useful for our first approach, clearly also restricts the generality of our results. Thus, in future studies it has to be shown which image features are predictive for the Likes in other types of photographs. In any case, the present paper provides a promising first step for such future research. Therefore, we conclude that Instagram Likes can be used as a proxy for aesthetic appeal and are indeed tied to objective features of the image, such as curvature and visual balance measures.

## Author contributions

KT: database collection, experimental design, data processing and analysis, drafting. RH: revising, computing the algorithms.

### Conflict of interest statement

The authors declare that the research was conducted in the absence of any commercial or financial relationships that could be construed as a potential conflict of interest.

## Footnotes

^1^4K Stogram: OpenMedia GmbH. Available online at: https://www.4kdownload.com/de/products/product-stogram

^2^To get an idea of the effect size, *Cohen's d* was calculated: *Cohen's d* = $\frac{M-\mu_{0}}{SD}$. Cohen (1988) outlined the following criteria for gauging small, medium and large effect sizes: small ≥ 0.20, medium ≥ 0.50, large ≥ 0.80.

^3^https://de.mathworks.com/help/matlab/ref/rgb2gray.html
